# An analysis of the structure and content of dashboards used to monitor patient safety in the inpatient setting

**DOI:** 10.1093/jamiaopen/ooab096

**Published:** 2021-11-17

**Authors:** Masha Kuznetsova, Michelle L Frits, Sevan Dulgarian, Christine Iannaccone, Elizabeth Mort, David W Bates, Hojjat Salmasian

**Affiliations:** 1 Harvard Business School, Boston, Massachusetts, USA; 2 Division of General Internal Medicine, Department of Medicine, Brigham and Women’s Hospital, Boston, Massachusetts, USA; 3 Department of Medicine, Massachusetts General Hospital, Boston, Massachusetts, USA; 4 Department of Health Care Policy, Harvard Medical School, Boston, Massachusetts, USA; 5 Mass General Brigham, Somerville, Massachusetts, USA

**Keywords:** data collection, patient safety, quality improvement, visualization

## Abstract

The objective of this study is to review and compare patient safety dashboards used by hospitals and identify similarities and differences in their design, format, and scope. We reviewed design features of electronic copies of patient safety dashboards from a representative sample of 10 hospitals. The results show great heterogeneity in the format, presentation, and scope of patient safety dashboards. Hospitals varied in their use of performance indicators (targets, trends, and benchmarks), style of color coding, and timeframe for the displayed metrics. The average number of metrics per dashboard display was 28, with a wide range from 7 to 84. Given the large variation in dashboard design, there is a need for future work to assess which approaches are associated with the best outcomes, and how specific elements contribute to usability, to help customize dashboards to meet the needs of different clinical, and operational stakeholders.

## INTRODUCTION 

Dashboards have been widely used by healthcare organizations to synthesize and help providers visualize large quantities of continuously updated data and to facilitate clinical, operational, and strategic decision-making. Visualizing key performance indicators (KPIs) in a dashboard format has been shown to decrease the time spent on collecting data, reduce the time to task completion, and alleviate cognitive load demands.^1–3^ Nevertheless, evidence regarding the effectiveness of dashboards in clinical and operational practice is limited. A review of clinical and quality dashboards found some evidence that presenting information in such a way is associated with better adherence to guidelines and a potential improvement in outcomes.^4^ Other studies found no significant impact on outcomes; for example, in one study, no significant association between dashboard use and preventive screening quality scores was identified.^5^ Importantly, much of the work in this literature involves case studies of single dashboards or review articles. These studies have generated important insights regarding development and implementation of dashboards in specific cases, but few comparative studies that contrast multiple dashboards across different institutions have been done.

Patient safety represents an area where effective dashboards can have a significant impact on outcomes by alerting decision-makers to potential issues and, thus, helping prevent harm.^6^ Clinical data elements that inform patient safety metrics can be siloed and not accessible to each type of provider, or recorded in multiple places within the electronic health record.^7^ By aggregating and presenting data in a single place, dashboards can play a key role in improving and monitoring patient safety. However, due to the sensitive nature of information presented in patient safety dashboards and organizations’ unwillingness to share this data with external researchers, research on patient safety dashboards has been limited.

Prior research has documented development, implementation, and utility of dashboards in several domains of patient safety including computerized provider-order entry, monitoring adverse events (eg, falls), and medication safety.^8–10^ But our previous research shows that organizations use safety measures in a much wider set of domains—for example, hospital acquired infections (HAIs), mortality and morbidity, surgical complications, and so on—which have not been subject to research focused on dashboards.^11^

Inherent tradeoffs exist between presenting the decision-maker with a comprehensive set of KPIs and information overload. Dashboard design, particularly for patient safety, requires a careful consideration of user experience and human factors engineering.^12^ Integration of such insights into the design of health information technology has been generally meager.^13^^,^^14^ Prior research documented issues related to the usability and provider experience with dashboards. Major drivers of worsened user experience include the layout, ease-of-use, and lack of clarity on the meaning of labels.^15^ Gaps in the design of dashboards may lead to unintended consequences associated with performance measurement, which can compromise their potential to improve rapid decision-making. These include tunnel vision, measure fixation (focusing on specific metrics rather than on the process of care), and misinterpretation of presented information.^4^^,^^16^

Improving patient safety and reducing avoidable harm is a key priority for health systems. Understanding the number and types of indicators an institution chooses to include in their patient safety dashboard(s) as well as the format in which they are presented can inform consistent design of patient safety dashboards and enhance safety measurement. Our objective was to review and compare patient safety dashboards used across a sample of hospitals to identify similarities and differences in their design, presentation, and purpose.

## MATERIALS AND METHODS

### Settings and data

This study was conducted as part of the Safe Care study, which is composed of several multi-center studies on safety measurement and quantification of patient harm. The project is supported by grant funding from CRICO, the liability insurer for Harvard-affiliated hospitals, and healthcare systems.

We obtained electronic copies of patient safety dashboards from 11 Harvard-affiliated healthcare delivery organizations. Ten offered both inpatient and outpatient services, while 1 only offered outpatient services and its dashboards exclusively captured outpatient safety events and non-acute indicators. To ensure a fair comparative analysis, we excluded the dashboards from the latter site and analyzed the data from the other 10 sites. The final sample included 3 academic medical centers and 7 community hospitals of varying size (2 large, 2 medium, and 3 small hospitals).

### Review process

The research team developed a data collection tool, which was used to review each dashboard. The tool is based on previous research on healthcare dashboards and included the following format and design elements: number and type of safety metrics present; display of benchmarks, targets, and trends; timeframe; platform the dashboard is presented on; and elements of visual design (eg, color coding).

In addition to presentation and specific visual elements, dashboards are informed by their purpose. To categorize dashboard purpose, Zhuang et al^17^ proposed a framework that builds on the idea of intended task, using a priori determinability and task complexity. A priori determinability assesses how easily the user can determine the process needed to complete a task before completing it.^18^ Task complexity assesses the amount of dashboard interactions the user needs to engage with to accomplish the task. Task complexity is low when the user can easily determine the status of each metric and required next steps, and vice versa. Based on where each dashboard lies on the spectrum of these 2 indicators, it is categorized as 1 of the 4 types: decision support, operational, tracking, or exploratory. A combination of analysis of visual characteristics and purpose can inform future evaluation of effectiveness of dashboards in practice and their role in the workflow. The present review assessed the level of a priori determinability and task complexity of each dashboard and categorized them by purpose.

For the sake of the analysis of design features, all dashboards from the same hospital were counted as 1. This was done because some sites used different tabs, pages, or files for each subset of KPIs and we did not want these choices of segmentation to influence our analysis of the contents of the dashboards. Furthermore, the design features (eg, color scheme) were consistent across different tabs within each hospital’s dashboard. In the analysis of the number of metrics displayed, dashboards from the same hospital were counted separately to capture the average number of metrics displayed, as well as the range.

Each dashboard was independently assessed by 2 reviewers, and any discrepancies were discussed until consensus. Data elements were summarized using descriptive methods reflecting the distribution of design and presentation elements across institutions in the sample.

## RESULTS

**Figure 1.**
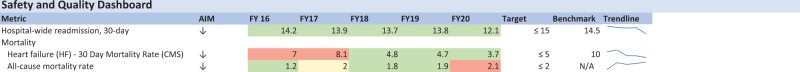
A schematic mock-up of elements of a safety dashboard. This mock-up demonstrates the use of trendlines, aims, benchmarks, and targets, as well as a red–green color coding of metric values.

Our analysis revealed substantial heterogeneity in the format and scope of patient safety dashboards (see Figure  1 for a schematic dashboard mock-up). The average number of metrics per dashboard display was 28, ranging from 7 to 84 (Table  1). Of the 10 dashboards, 9 used color coding to visually denote whether performance was positive or negative. However, the color schemes varied across the systems. Only 2 (2/10) dashboards provided a comprehensive set of definitions for the metrics displayed.

**Table 1.** Summary of dashboard features

Dashboard feature	Number of dashboards that included the feature (%), *N* = 10
Visual display	
Color coding	9 (90)
Color coding legend	7 (70)
Definitions	2 (20)
Performance status	
Benchmarks	5 (50)
Targets	5 (50)
Trendlines	6 (60)
Aim	7 (70)
Timeframe	
Monthly updates	2 (20)
Quarterly updates	5 (50)
Annual updates	3 (30)
Audience	
Internal monitoring authority	1 (10)
Platform	
Tableau	4 (40)
Excel	2 (20)
Midas	2 (20)
Google sheets	1 (10)
Excel + Tableau	1 (10)

There was also variation within subgroups of hospitals (Table  2). For example, of the 3 academic hospitals, 2 used explicit targets for each metric while 1 used a combination of benchmark and trendline. Similarly, among the 7 community hospitals, 3 used explicit targets, and 4 others provided either a benchmark or a benchmark with trendline. The average number of indicators per dashboard display was 33 for community and 21 for academic hospitals, although the ranges varied widely within each type. Similar within-group heterogeneity was recorded when comparing hospitals by size.

**Table 2.** Comparison of select dashboard features by hospital type and size

Dashboard feature	Hospital type		Hospital size	
	Academic (*N* = 3)	Community (*N* = 7)	Large (*N* = 5)	Small, medium (*N* = 5)
Color coding	3/3	6/7	4/5	5/5
Explicit target	2/3	3/7	3/5	2/5
Trendline	2/3	4/7	2/5	4/5
Annual updates	1/3	2/7	2/5	1/5
Quarterly updates	2/3	3/7	3/5	2/5
Monthly updates	0/3	2/7	0/4	2/5
Mean number of indicators per dashboard display	21 (range: 7–72)	33 (range: 10–84)	24 (range: 7–72)	33 (range: 10–84)

In terms of their purpose, most dashboards in the sample were categorized as operational (9/10), and only 1 (1/10) was primarily designed for decision support. Operational dashboards ranked low on task complexity and high on a priori determinability. Their goal is to visualize the status of KPIs and required actions at a glance, involving minimal cognitive load.^17^ As such, all operational dashboards in our sample included performance status indicators. However, among them, there was heterogeneity in how the status of each metric was presented. Of 9 operational dashboards, 4 had explicit targets, 5 had performance benchmarks, and 5 included trendlines that signaled the direction of performance.

**Figure 2.**
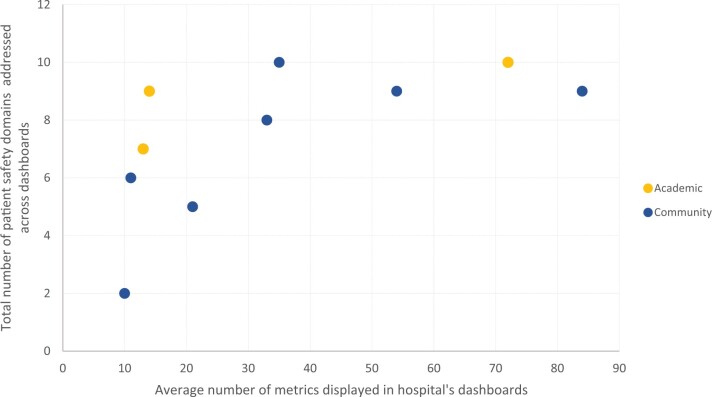
Relationship between the average number of indicators per dashboard displayed and the total number of patient safety domains covered in each hospital’s dashboards. Each point represents a hospital in the sample.

**Figure 3.**
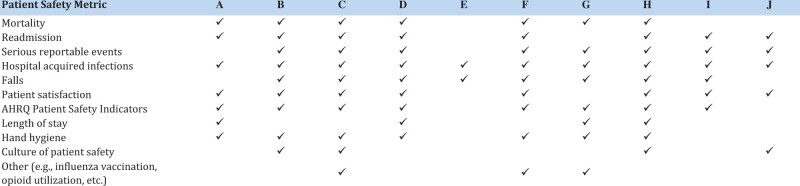
Domains of patient safety metrics included in each hospital’s (hospitals A–J) dashboards.

Consistent with prior literature, hospitals differed in terms of the types of patient safety metrics presented.^11^ The level of detail provided for each metric differed as well. For example, although all hospitals presented measures of HAIs, there was variation in the extent of specific infections displayed. A higher average number of metrics did not consistently correspond to more patient safety domains covered, and vice versa (Figure  2). Some dashboards included metrics that may not be viewed as safety metrics by all, such as process measures on hand hygiene, results of culture of safety surveys, or outcomes such as readmission (Figure  3).^19^

## DISCUSSION

We compared patient safety dashboards from 10 hospitals and found substantial heterogeneity in both their scope and design. High variability in the contents of these dashboards implies lack of conformity in the hospitals’ approach to safety measurement. While some of the variability might arise from customizing displays to different audiences, it is likely that some stems from a lack of established best practices in design and user experience standards. We acknowledge that a well-designed dashboard will not result in improvement unless used effectively in the context of an organization’s quality and safety improvement framework. Dashboards will likely be critical, though, for improving safety, and represent an important foundational tool to elevate the importance of patient safety and help with prioritizing improvement efforts and tracking progress.

We found large variation in the format of dashboards, approaches to visualizing the data, and presence of targets or benchmarks. Certain design aspects seemed to be influenced by the choice of platform used to deliver the dashboards; for example, some platforms enforce color pallets that work best for individuals with color blindness or visual impairment (eg, using blue and orange to indicate good and bad outcomes, respectively) and dashboards that did not use these platforms generally lacked a similar design consideration. In line with prior work, the present review points to the need to better understand how specific dashboard characteristics contribute to outcomes.^4^

The variation in the number of indicators could be related to their intended audience. For example, if a dashboard is intended for multiple stakeholders with varying needs, it may include a higher number of indicators. This would allow all intended users to have access to all the information, with the expectation that each group would focus on the relevant subset. Nevertheless, the information load caused by dashboards with a larger number of metrics may interfere with their effectiveness, and the fact that different dashboards from the same organization had substantial variability may suggest that information load implications are not consistently considered in dashboard design. The variability revealed in this review emphasizes the need to incorporate human factors engineering in dashboard design and implementation of patient safety monitoring systems.^12^ Considering the impact of information load on different user groups is key to ensure that data visualization achieves its full potential and supports effective decision-making. Furthermore, it is important to understand how the scope and design elements contribute to potential unintended consequences, such as measure fixation, tunnel vision, and interpretation challenges.^15^^,^^16^

The present study had several limitations. Our review focused on evaluating the format, visual tools, and purpose of patient safety dashboards. Given the cross-sectional design of the study, we were not able to assess outcomes associated with each dashboard. Our study included dashboards from 10 hospitals affiliated with the same medical school. This allowed us to demonstrate that even related hospitals have heterogeneous dashboards and to study the relationship of this variability with respect to hospital characteristics; however, a similar study on dashboards from completely independent hospitals may improve generalizability of the results. Finally, because we used electronic copies of the dashboards, we were unable to study usability or utilization by user group. These are important questions for future research.

With abundance of data, effective presentation of key data trends is critical. As dashboards allow the viewer to see the most critical data points in a snapshot to inform decision-making, it is important to identify the most effective ways of displaying data, implement them in practice, and use empirical methods to evaluate the results. While multiple views will likely be useful, standardizing presentation across units and facilities will make comparisons and tracking of trends easier. To maximize the utility of patient safety dashboards, future work is necessary to understand how specific features of dashboards relate to their effectiveness and outcomes. Given the variation in the number of metrics and scope of patient safety dashboards, generating evidence to customize dashboards to specific clinical and operational audiences remains important.

## FUNDING

The work was supported by RMF, but no CRICO/RMF employee was an author or provided help and guidance in conducting the research.

## AUTHOR CONTRIBUTIONS

MK and HS conceived the study and drafted the manuscript. MLF, SD, and CI collected the data and assisted in conducting the review process. EM and DWB provided input regarding interpretation of findings. All authors contributed to revising the manuscript and approved its submission.

## CONFLICT OF INTEREST STATEMENT

DWB reports grants and personal fees from EarlySense, personal fees from CDI Negev, equity from ValeraHealth, equity from Clew, equity from MDClone, personal fees and equity from AESOP, personal fees and equity from Feelbetter, and grants from IBM Watson Health, outside the submitted work. All other authors do not have competing interests to declare.

## DATA AVAILABILITY

The data used in this article will be shared on reasonable request.
